# The Iron-Dependent Regulation of the *Candida albicans* Oxidative Stress Response by the CCAAT-Binding Factor

**DOI:** 10.1371/journal.pone.0170649

**Published:** 2017-01-25

**Authors:** Ananya Chakravarti, Kyle Camp, David S. McNabb, Inés Pinto

**Affiliations:** 1 Department of Biological Sciences, University of Arkansas, Fayetteville, Arkansas, United States of America; 2 Cell and Molecular Biology Program, University of Arkansas, Fayetteville, Arkansas, United States of America; Newcastle University, UNITED KINGDOM

## Abstract

*Candida albicans* is the most frequently encountered fungal pathogen in humans, capable of causing mucocutaneous and systemic infections in immunocompromised individuals. *C*. *albicans* virulence is influenced by multiple factors. Importantly, iron acquisition and avoidance of the immune oxidative burst are two critical barriers for survival in the host. Prior studies using whole genome microarray expression data indicated that the CCAAT-binding factor is involved in the regulation of iron uptake/utilization and the oxidative stress response. This study examines directly the role of the CCAAT-binding factor in regulating the expression of oxidative stress genes in response to iron availability. The CCAAT-binding factor is a heterooligomeric transcription factor previously shown to regulate genes involved in respiration and iron uptake/utilization in *C*. *albicans*. Since these pathways directly influence the level of free radicals, it seemed plausible the CCAAT-binding factor regulates genes necessary for the oxidative stress response. In this study, we show the CCAAT-binding factor is involved in regulating some oxidative stress genes in response to iron availability, including *CAT1*, *SOD4*, *GRX5*, and *TRX1*. We also show that *CAT1* expression and catalase activity correlate with the survival of *C*. *albicans* to oxidative stress, providing a connection between iron obtainability and the oxidative stress response. We further explore the role of the various CCAAT-binding factor subunits in the formation of distinct protein complexes that modulate the transcription of *CAT1* in response to iron. We find that Hap31 and Hap32 can compensate for each other in the formation of an active transcriptional complex; however, they play distinct roles in the oxidative stress response during iron limitation. Moreover, Hap43 was found to be solely responsible for the repression observed under iron deprivation.

## Introduction

*Candida albicans* exists as a commensal in healthy individuals; however, it is capable of causing infections ranging from superficial mucosal to systemic life threatening infections in immunocompromised individuals [1,2]. While the virulence of *C*. *albicans* is multifactorial, one necessary aspect of survival is the ability to survive the host immune response. By contrast, innate immune cells, such as macrophages and neutrophils, attempt to kill invading pathogens by exposing them to superoxides, peroxides, and hydroxyl radicals, collectively called Reactive Oxygen Species (ROS), through a process called the respiratory burst [3–7]. *C*. *albicans* defends against the respiratory burst by expressing an array of antioxidant enzymes such as catalase, superoxide dismutases, glutaredoxins and thioredoxins [4,8–12].

While our current understanding of the Oxidative Stress Response (OSR) developed through the study of various yeast and fungi, including *Saccharomyces cerevisiae*, the response in *C*. *albicans* displays distinct differences in the regulatory circuits that govern the stress response across fungal clades [13,14]. For example, *C*. *albicans* lacks the general Msn2/Msn4-mediated stress response and the cross protection to different stresses is poor to negligible, unlike the response seen in *S*. *cerevisiae* or *Schizosaccharomyces pombe* [14,15]. Similarly, the Stress Activated Protein Kinase (SAPK), Hog1, functions in osmotic stress in both *S*. *cerevisiae* and *C*. *albicans*; however, it is also involved in the OSR in *C*. *albicans* [16–19]. Even within the same genus, *C*. *albicans* and *C*. *glabrata* have strikingly different regulatory mechanisms for dealing with oxidative stress [4].

The genes involved in the OSR are conserved among fungal pathogens and benign model yeasts. *C*. *albicans* has a single gene encoding catalase (*CAT1*) that converts hydrogen peroxide to water and oxygen [20]. *C*. *albicans* encodes six distinct superoxide dismutases, with *SOD2* and *SOD3* being homologous to the Mn-Sod family while *SOD1*, *SOD4*, *SOD5*, and *SOD6* are homologous to the CuZn-Sod family [21,22]. More recently it was demonstrated that Sod5 is a unique Cu-only superoxide dismutase [9]. These enzymes convert superoxide anions to hydrogen peroxide which is further processed by catalase to water and oxygen. In addition, *C*. *albicans* encodes four putative glutaredoxins, *GRX1*, *GRX2*, *GRX3* and *GRX5* and two thioredoxins, *TRX1* and *TRX2* [23,24]. With the large array of proteins involved in the OSR, it is likely that a subset of these antioxidant enzymes will be coordinately regulated to facilitate the survival of *C*. *albicans* in the dynamic micro-niches of the host.

The expression of some antioxidant enzymes appears to be regulated by iron availability [25,26]. Iron poses an interesting dilemma for cells since it is essential for the activity of many enzymes, yet an excess of intracellular iron can catalyze the formation of reactive oxygen species, via the Fenton reaction, resulting in oxidative cell damage [27]. Thus, the maintenance of intracellular iron homeostasis is essential for normal growth and minimizing the oxidative damage associated with iron overload. For an invading pathogen, the human host is essentially a low-iron environment with limited free iron [28,29]. To combat the iron sequestration by the host, *C*. *albicans* has evolved multiple mechanisms to acquire iron that involve a reductive uptake mechanism, a siderophore scavenging pathway and a hemoglobin uptake pathway [30–32].

In *C*. *albicans*, several transcription factors have been found to be involved in iron acquisition/utilization pathways [30,31,33]. One of these transcription factors, the CCAAT-binding factor, has been shown to be important for virulence [25,26,34]. Work from our lab has previously demonstrated that the CCAAT-binding factor is important for the regulation of genes involved in respiratory metabolism, namely *CYC1* and *COX5a*, in response to carbon source availability [35]. Moreover, it has been observed that the CCAAT-binding factor is important for the regulation of genes involved in acquisition/utilization of iron [25,26,34,36,37]. Since the generation of reactive oxygen species in actively growing cells occurs via the Fenton reaction or as a byproduct of respiratory metabolism, we hypothesized that the CCAAT-binding factor may play a central role in regulating the OSR, thereby coordinately regulating iron acquisition/utilization, respiratory metabolism, and the OSR. In fact, whole genome microarray expression studies performed by Singh et al. [34] suggested that genes involved in the OSR were regulated by the CCAAT-binding factor.

The CCAAT-binding factor is an evolutionarily conserved heterooligomeric transcription factor that binds to the consensus 5’-CCAAT-3’ sequence in the promoters of target genes [38]. In *S*. *cerevisiae*, the CCAAT-binding factor is composed of three subunits, Hap2, Hap3, and Hap5, necessary for DNA-binding and a fourth subunit, Hap4, the effector subunit responsible for transcriptional activation [39–41]. It has been well-characterized in *S*. *cerevisiae* as the activator of genes involved in respiratory metabolism as well as other pathways [41–45]. In *C*. *albicans*, the CCAAT-binding factor is comprised of the Hap2 and Hap5 subunits; however, there are two distinct homologs of Hap3, termed Hap31 and Hap32 [34,35,37]. In addition, there are three putative homologs of Hap4, termed Hap41, Hap42, and Hap43 [34,35]. Previous studies have shown that deletion of either *HAP2* or *HAP5* leads to complete abolishment of the DNA-binding activity in both *S*. *cerevisiae* and *C*. *albicans* [35,36,40]. In *C*. *albicans* it is plausible that the Hap31 and Hap32 may individually interact with Hap2 and Hap5 to form DNA-binding complexes with differing regulatory functions via interaction with the three Hap4-like proteins [34].

In the experiments presented herein, we show that the CCAAT-binding factor is involved in the iron-dependent differential expression of *CAT1* in *C*. *albicans*. We further provide evidence to support a role for the CCAAT-binding factor in the iron-dependent regulation of other genes involved in the OSR, including *SODs*, *GRX*s and *TRXs*. We used *CAT1* as the prototype gene to ask whether the differential regulatory pattern is achieved, at least partially, through the iron-dependent recruitment of distinct CCAAT-binding factor complexes to target promoters. Lastly, we discuss a framework for the role of the CCAAT-binding factor in the iron-dependent transcriptional regulation of the OSR.

## Materials and Methods

### Yeast strain and growth conditions

The yeast strains used in this study are listed in S1 Table. Strains were routinely cultured in yeast extract-peptone-dextrose (YPD) medium [46]. For DNA transformations, synthetic complete (SC) medium lacking auxotrophic supplements or synthetic minimal medium (SD) augmented with the auxotrophic requirements was used [46]. To generate iron depleted growth conditions, bathophenanthroline disulfonate (BPS) (Sigma) was added to the growth medium at the indicated concentration.

### Oligonucleotides and plasmid construction

The oligonucleotides used in this study are listed in S2 Table. The plasmid pDM588 containing *HAP41* was generated by PCR amplification of *C*. *albicans* orf19.740 using the oligonucleotide primers oDM0343/oDM0344. The *HAP41* PCR product was digested with BamHI/PstI cloned into the same sites of pSP65 (Promega Corp). Plasmid pDM588 was digested with NdeI/ClaI, the ends blunted with T4 polymerase and a BglII linker ligated to generate pDM589 containing a deletion in the *HAP41* coding sequence. To generate pDM592 with the *hap41*::*URA3* allele, *URA3* was amplified from pGEM-URA3 [47] using primers oDM0382/oDM0383 that generated BamHI sites on both the 5’ and 3’ ends of the gene. Plasmid pDM589 and the *URA3* PCR product were digested with BglII and BamHI, respectively, and ligated. To generate pDM598 containing the *hap41*::*HIS1* allele, *HIS1* was amplified from pGEM-HIS1 [47] with primers oDM0384/oDM0385 that generated BclI sites on both the 5’ and 3’ ends of the gene. Plasmid pDM589 and the *HIS1* PCR product were digested with BglII and BclI, respectively, and ligated. The plasmid pDM571 containing *HAP42* was generated by PCR amplification of the *C*.*albicans* orf19.1481 with the oligonucleotide primers oDM0345/oDM0346 that incorporated unique BamHI and HindIII restriction sites into the 5’ and 3’ ends of the gene, respectively. The plasmid YEplac181 and the *HAP42* PCR product were digested with BamHI/HindIII and ligated to generate pDM571. To create *hap42Δ*::*hisG-URA3-hisG* knockout allele, pDM571 was digested with BamHI/HindIII and *HAP42* was ligated into pSP65 digested with BamHI/HindIII to generate pDM800. The plasmid pDM800 was subsequently amplified by PCR using primers oDM0588/oDM0589 that yielded a plasmid product with a deletion of the *HAP42* coding region, but retaining approximately 500bp of flanking sequence and a unique BglII restriction site on the 5’ and 3’ ends. The DNA was digested with BglII and ligated with the BamHI/BglII digested *hisG-URA3-hisG* from the plasmid p5921 [48]. The plasmid pDM602 contains *HAP43* that was generated by PCR amplification of the *C*. *albicans* orf19.681 with the oligonucleotide primers oDM0394/oDM0395 that incorporated unique BamHI and SalI restriction sites. The BamHI/SalI digested PCR product was cloned into BamHI/SalI digested pSP65 to generate pDM801. The plasmid pDM801 was used as a template for PCR with primers oDM0590/oDM0591 that created a plasmid product with a deleted coding sequence, but containing approximately 1000bp of *HAP43* flanking sequence and a unique BglII restriction site on the 5’ and 3’ ends. The DNA was digested with BglII, ligated with the BamHI/BglII digested *hisG-URA3-hisG* from the plasmid p5921 [48] and the plasmid designated pDM803. For construction of pDM802 containing the *CAT1* promoter fused to *Renilla* luciferase (*Rluc*), a 1000bp region of *CAT1*, upstream of the start codon of orf19.13609, was amplified by PCR using the oligonucleotide primers oDM0660/oDM0661, which incorporated unique SphI and BamHI sites into the 5’ and 3’ ends of the PCR product, respectively. The *CAT1* promoter fragment was digested with SphI/BamHI and cloned into the SphI/BamHI sites of pDM692.

### Construction of *C*. *albicans* strains

All DNA transformation procedures were performed using the lithium acetate transformation kit (QBiogene, Inc.) per the manufacturer instructions. All of the strains described are isogenic derivatives of BWP17 except for the indicated *hap* allele under study. We have noted BWP17-derived strains carry a mutation in *IRO1*, a gene adjacent to *URA3* locus that has been implicated in iron utilization ([49,50]. However, any potential effect of *iro1* in our study would be the same for all strains. The construction of the *hap2Δ/Δ* (DMC249), *hap31Δ/Δ* (DMC280), *hap32Δ/Δ* (DMC285) and the *hap31Δ/Δ hap32Δ/Δ* (DMC290) mutants have be described elsewhere [51]. The *hap41Δ/Δ* mutant (DMC190) was generated by two consecutive rounds of transformation of the parent strain BWP17 using the *hap41Δ*::*URA3* and *hap41Δ*::*HIS1* disruption cassettes. The *hap41Δ*::*URA3* was released from plasmid pDM592 by digestion with BamHI/HindIII, introduced into BWP17, and selected on SC-Ura medium. To verify the *HAP41/hap41Δ*::*URA3* heterozygote, genomic DNA was isolated from transformants as described previously [52] and PCR was used to confirm the appropriate recombination. For PCR the oligonucleotide primers oDM0369 (anneals within the *URA3* gene) and oDM0620 (anneals to *HAP41* locus upstream of the recombination) were used. The *HAP41/hap41Δ*::*URA3* heterozygote was subsequently transformed with BamHI/HindIII-digested pDM598 containing the *hap41Δ*::*HIS1* allele and transformants were selected on SC-His medium. The transformants were subsequently tested on SC-His-Ura medium to verify knockout of both loci. Genomic DNAs were prepared from His^+^ Ura^+^ transformants and PCR was used to verify the correct recombination, using oligonucleotide primers oDM0370 (anneals within *HIS1*) and oDM0620. The *hap42Δ/Δ* mutant DMC350 was generated as follows. *C*. *albicans* BWP17 was subjected to two consecutive rounds of DNA transformation with the *hap42Δ*::*hisG-URA3-hisG* cassette after release of the knockout cassette from pDM800 by digestion with BamHI/HindIII. Transformants were selected on SC-Ura medium. To confirm deletion of the first copy of *HAP42*, genomic DNA was isolated and PCR was performed with oligonucleotides primers oDM0617 (anneals to *HAP42* locus upstream of the recombination) and oDM0369 (anneals within *URA3*). Following confirmation, the *HAP42/hap42Δ*::*hisG-URA3-hisG* heterozygote was grown on 5-fluoroorotic acid (5-FOA) medium to select from Ura^-^ recombinants. The transformation was repeated for the deletion of the second allele of *HAP42* and the *hap42Δ/Δ* mutant confirmed by PCR with same oligonucleotide primers. The *hap43Δ/Δ* strain DMC351 was generated in a similar manner using the *hap43Δ*::*hisG-URA3-hisG* cassette after release of the cassette from pDM803 by digestion with BamHI and SalI. The gene disruptions were verified using oligonucleotide primers oDM0396 (anneals to the *HAP43* locus upstream of recombination) and oDM369 (anneals within *URA3*). The *hap42Δ/Δ hap43Δ/Δ* double mutant (DMC352) was constructed by disrupting *HAP43* using the *hap43Δ*::*hisG-URA3-hisG* in the *hap42Δ/Δ* strain DMC350. The *hap41Δ/Δ hap42Δ/Δ* (DMC353) and the *hap41Δ/Δ hap43Δ/Δ* (DMC354) double mutants were generated from the *hap42Δ* (DMC350) and *hap43Δ* (DMC351) strains, respectively, by transformation with the *hap41Δ*::*URA3* and the *hap41Δ*::*HIS1* disruption constructs sequentially and the resulting disruption was confirmed by PCR as outlined above. The *hap41Δ/Δ hap42Δ/Δ hap43Δ/Δ* triple mutant (DMC355) was generated using the *hap42Δ/Δ hap43Δ/Δ* strain DMC352 and disrupting the *HAP41* with *hap41Δ*::*URA3* and the *hap41Δ*::*HIS1* disruption constructs sequentially as described above. All of the strains were confirmed by Southern blot as previously described [35]. *C*. *albicans* strains expressing the *CAT1-Rluc* reporter pDM802 were generated by linearizing pDM802 with HpaI within *ARG4* and introducing the plasmid into the appropriate strains (BWP17 and DMC108) containing the *arg4* auxotropy yielding the strains DMC356 and DMC357, respectively. The resulting transformants were selected on SC-Arg and at least three independent colonies were used for the luciferase assays. After strain construction, the remaining auxotrophies were rescued. For *arg4*, the plasmid pDM583 was linearized with HpaI within *ARG4* and introduced into the appropriate strains. For the *ura3* and *his1*, the plasmid pDM605 (containing *URA3* and *HIS1*) was linearized with NruI within *HIS1* and introduced into the appropriate strains. The final prototrophic strains were confirmed by growth on synthetic minimal medium.

### Northern blot analysis

*C*. *albicans* strains were grown to saturation in YPD for iron replete conditions or YPD + 200 μM BPS for iron-limiting conditions, and subsequently reinoculated into the respective medium and grown to an OD_600nm_ of 0.5–0.8 at 30°C. The cells were harvested by centrifugation, and total RNA was prepared by the glass bead-acid phenol method as previously described [53]. Approximately 20 μg of total RNA was loaded, separated by formaldehyde-1% agarose gel electrophoresis, and transferred to GeneScreen Plus membranes (Dupont-NEN Research products) according to manufacturer’s protocol. The membranes were hybridized and washed under standard high-stringency conditions [35]. The *CAT1* and 26S *rRNA* probes were obtained by PCR amplification from *C*. *albicans* BWP17 genomic DNA using the following primer pair oDM0621/oDM0622 and oDM0459/oDM0460, respectively. The *SOD1*, *SOD2*, *SOD3*, *SOD4*, *SOD5*, *and SOD6* probes were obtained by PCR amplification using the following primer pair oDM0650/oDM0651, oDM0626/oDM0627, oDM0628/oDM0629, oDM0652/oDM0653, oDM0654/oDM0655 and oDM0656/oDM0657, respectively. The *GRX2*, *GRX3*, *GRX5* and *TRX1* probes were obtained by PCR amplification using oligonucleotide pairs oDM0665/oDM0666, oDM0632/oDM0633, oDM0630/oDM0631 and oDM0634/oDM0635, respectively. The probes were purified by agarose gel electrophoresis and GeneClean (Qbiogene, Inc.), and radiolabeled with [α-^32^P] dATP (MP Biomedicals, LLC) by a random primer labelling kit (U.S. Biochemicals) according to the manufacturer’s protocol. The transcript levels were quantified on a model 9600 Typhoon imager (GE Healthcare Life Sciences, Piscataway, NJ). All Northern blotting experiments shown are representative of at least two, and most cases three, independent experiments from different total RNA preparations.

### Catalase enzymatic assays

Catalase activity was determined by monitoring the decomposition of hydrogen peroxide spectrophotometrically at 240nm as previously described [54]. The cells were grown to saturation in YPD or YPD + 200 μM BPS medium, the cultures were subsequently diluted in YPD or YPD + 150 μM BPS and grown to an OD_600nm_ of 0.5 to 0.8. The cells were harvested by centrifugation at 14,000 X *g*, washed with water and the cell pellets weighed. The pellet was suspended in 50 mM potassium phosphate buffer (pH 7.2) containing 0.2 mM phenylmethylsulfonylfluoride (PMSF) such that the final cell concentration was 0.25 gm of cells/ml wet weight. The cells were disrupted using the Mini-Beadbeater (Biospec products) in the presence of 0.5 mm glass beads and subsequently centrifuged at 14,000 X *g* to obtain cell free lysate for the catalase assay. The total protein concentration of the lysates was determined by the Bradford’s protein assay (Biorad). For the catalase activity assay, the Beckman-Coulter DU 800 spectrophotometer was zeroed using 50 mM potassium phosphate buffer containing 40 μl of the cell lysate. Following the addition of 400 μl of 30% w/w hydrogen peroxide (Sigma), the decomposition of hydrogen peroxide was determined by measuring the continuous decrease in absorbance at 240 nm and the activity was calculated from the linear range of the curve. The catalase activity of each strain was proportional to the amount of hydrogen peroxide decomposed as determined by: Δμmol H_2_O_2_/min./μg cell lysate _=_ ΔA_240_ /(1.5min X 39.7m^-1^cm^-1^ X 1000 X μg cell lysate), where the Δμmol H_2_O_2_ is the change in micromoles of hydrogen peroxide per min per microgram lysate, ΔA_240_ is the change in absorbance at 240nm, 39.7m^-1^cm^-1^ is the molar extinction coefficient of hydrogen peroxide at 240 nm.

### Hydrogen peroxide sensitivity assays

Each strain was grown to saturation in YPD or YPD + 200 μM BPS medium, the cultures were subsequently diluted in YPD or YPD + 150 μM BPS, respectively, and grown to an OD_600nm_ of 0.5 to 0.8. The cells were harvested by centrifugation for 1 min. at 14,000 X *g*, washed twice with sterile deionized water, and quantified using a hemocytometer. Approximately 1 x 10^7^ cells of each strain were suspended in YPD medium containing 0, 40, and 80 mM hydrogen peroxide and incubated for 2 h at 30°C. To assess the hydrogen peroxide sensitivity, ten-fold serial dilutions were plated on YPD medium and incubated at 30°C.

### Renilla luciferase assays

*Renilla* Luciferase assays were performed using the *Renilla* Luciferase reporter assay system (Promega Corp., Madison, WI). For the luciferase measurements, all yeast strains were grown overnight in YPD medium with or without 100 μM BPS for iron replete or iron-limiting growth, respectively. The cultures were subsequently diluted and grown to mid-log phase in YPD or YPD + 150 μM BPS at 30°C. The cell density of the cultures was determined by absorbance at A_600nm_. A 1 ml aliquot of each culture was removed and centrifuged at 14,000 rpm for one min. The supernatant was removed and the cells were resuspended on 100 μl of 1X lysis buffer (Promega Corp.), and sterile glass beads were added. The samples were vortexed for one min, cooled on ice for 30 seconds, and vortexed for another one min. The samples were centrifuged for one minute at 14,000 rpm to clarify the lysate. For the luciferase assay, 10 μl of cell lysate was added to a luminometer tube along with 100μl of *Renilla* luciferase substrate and luminescence was measured using a Turner Designs TD-20/20 luminometer. The final *Renilla* Luciferase activity was calculated with the following formula: *RLA* = *RLU/OD X (Va X Vc/Vb)*, where RLA is Relative Luciferase Activity in arbitrary units, RLU is the *Renilla* Luciferase luminescence determined by luminometry, OD is optical density of the cell culture at A_600nm_, Va is the volume of lysate used in the assay (0.01 ml), Vb is volume of lysis buffer (0.1 ml), Vc is volume taken from original culture (1 ml).

## Results

### The CCAAT-binding factor is a transcriptional regulator of *CAT1* in response to iron

**Fig 1:**
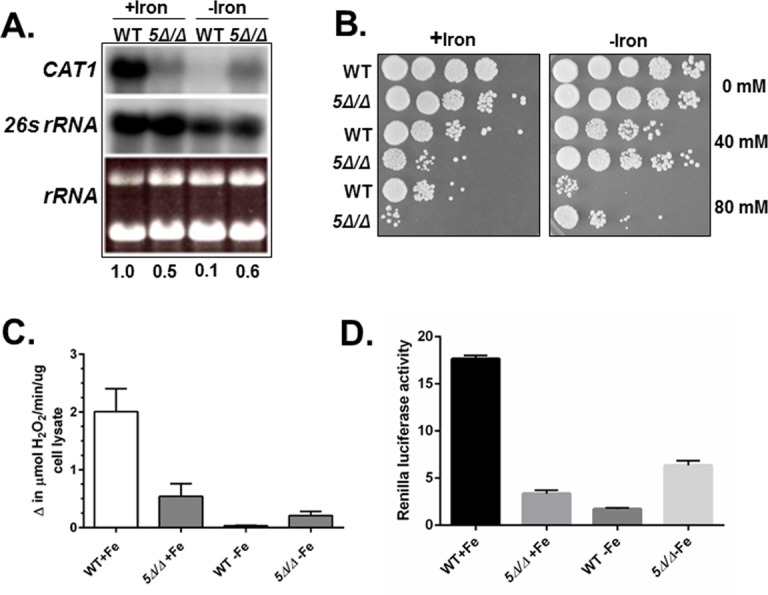
The CCAAT-binding factor regulates *CAT1* in response to iron availability. **(A)** Northern blot analysis of *CAT1* mRNA expression in the wild-type (DMC146) and *hap5Δ/Δ* mutant (DMC117) following growth in iron-replete (+iron) and iron-limiting (-iron) medium. The 26s rRNA was the loading control. mRNA levels were normalized to the 26s rRNA control using the WT as the reference value. **(B)** *C*. *albicans* wild-type (DMC146) and *hap5Δ/Δ* mutant (DMC117) were grown in iron-replete (+iron) or iron-limited (-iron) medium and subsequently exposed to hydrogen peroxide at the indicated concentrations for 2 h at 30°C. Ten-fold serial dilutions were spotted to YPD medium and incubated at 30°C for 3 days. **(C)** Catalase enzymatic activity in cell extracts derived from the wild type (DMC146) and *hap5Δ/Δ* mutant (DMC117) following growth in iron-replete (+iron) and iron-limiting (-iron) medium. The enzymatic assays are the average of three independent experiments with the error bars indicating the standard error. **(D)** *C*. *albicans* wild-type (DMC356) and *hap5Δ/Δ* mutant (DMC357) were grown in iron-replete (+iron) or iron-limited (-iron) medium and subsequently assayed for expression of *Renilla* luciferase driven by the *CAT1* promoter. The luciferase assays are the average of three independent experiments with the error bars indicating the standard error.

Since the *C*. *albicans* CCAAT-binding factor has been suggested to function in both transcriptional activation and repression of genes involved in respiratory metabolism and iron homeostasis [34–36], we hypothesized some OSR genes may be coordinately regulated by the same transcription factor to protect cells from reactive oxygen species generated during respiratory metabolism or by iron via the Fenton reaction. To test this hypothesis, we examined the level of *CAT1* mRNA in the wild-type versus *hap5Δ/Δ* mutant after growth in iron replete (YPD) and iron-limiting (YPD+BPS) medium. Total mRNA was isolated from the strains and Northern blots performed. As shown in Fig 1A, the *hap5Δ/Δ* strain showed a 4- to 5-fold decrease in *CAT1* mRNA levels compared to the wild-type strain after growth in iron replete medium. In contrast, when cells were grown in iron-limiting medium, the *hap5Δ/Δ* strain showed significantly higher expression of *CAT1* than the wild-type strain. These data suggest that the CCAAT-binding factor acts as a transcriptional activator in iron replete medium and as a transcriptional repressor during iron limitation. Alternatively, we cannot exclude the indirect possibility of the CCAAT-binding factor activating an as yet unidentified repressor during iron-limited growth. To determine whether the difference in mRNA levels had observable consequences at the phenotypic level, we examined the sensitivity of wild-type and *hap5Δ/Δ* strains to oxidative stress induced by hydrogen peroxide after the initial growth in iron replete (YPD) versus iron-limiting (YPD + BPS) medium. As shown in Fig 1B, the *hap5Δ/Δ* strain is more sensitive to oxidative stress after growth in iron replete medium; whereas, the *hap5Δ/Δ* mutant displayed more resistance to oxidative stress following growth under iron limitation. The phenotype was consistent with the level *CAT1* mRNA observed in the *hap5Δ/Δ* mutant; however, it was plausible that the mRNA levels may not reflect the catalase enzymatic activity in the cells. This is particularly relevant because catalase contains porphyrin heme groups; therefore, the enzymatic activity may be absent due to iron depletion. To examine this possibility, we measured catalase activity from cell extracts prepared from wild-type and *hap5Δ/Δ* mutant after growth in iron replete and iron-limiting medium. As shown in Fig 1C, catalase activity was qualitatively similar to the *CAT1* mRNA levels, with higher activity in the wild type versus the *hap5Δ/Δ* strain when cells were grown in iron replete medium. Under iron limitation, catalase activity was substantially reduced; however, the activity was reproducibly higher in the *hap5Δ/Δ* strain, suggesting the increased resistance to hydrogen peroxide stress (Fig 1B) was due to the increase in catalase activity. To demonstrate that the CCAAT-binding factor is regulating *CAT1* expression at the transcriptional level, a *CAT1-Renilla* luciferase reporter plasmid was introduced into a wild-type and *hap5Δ/Δ* mutant and luciferase activity was determine after growth in iron-replete and iron-limiting conditions. As shown in Fig 1D, the *Renilla* luciferase activity observed from the *CAT1-Rluc* reporter supported the Northern blot data (Fig 1A), indicating that mRNA stability is not involved in the levels of *CAT1* expression we observed. It is important to note that we have not demonstrated directly CCAAT-binding factor binding to the putative CCAAT sites within the *CAT1* promoter; therefore, it remains plausible that the CCAAT-binding factor indirectly influences *CAT1* mRNA expression.

### Regulation of other OSR genes by the CCAAT-binding factor

**Fig 2:**
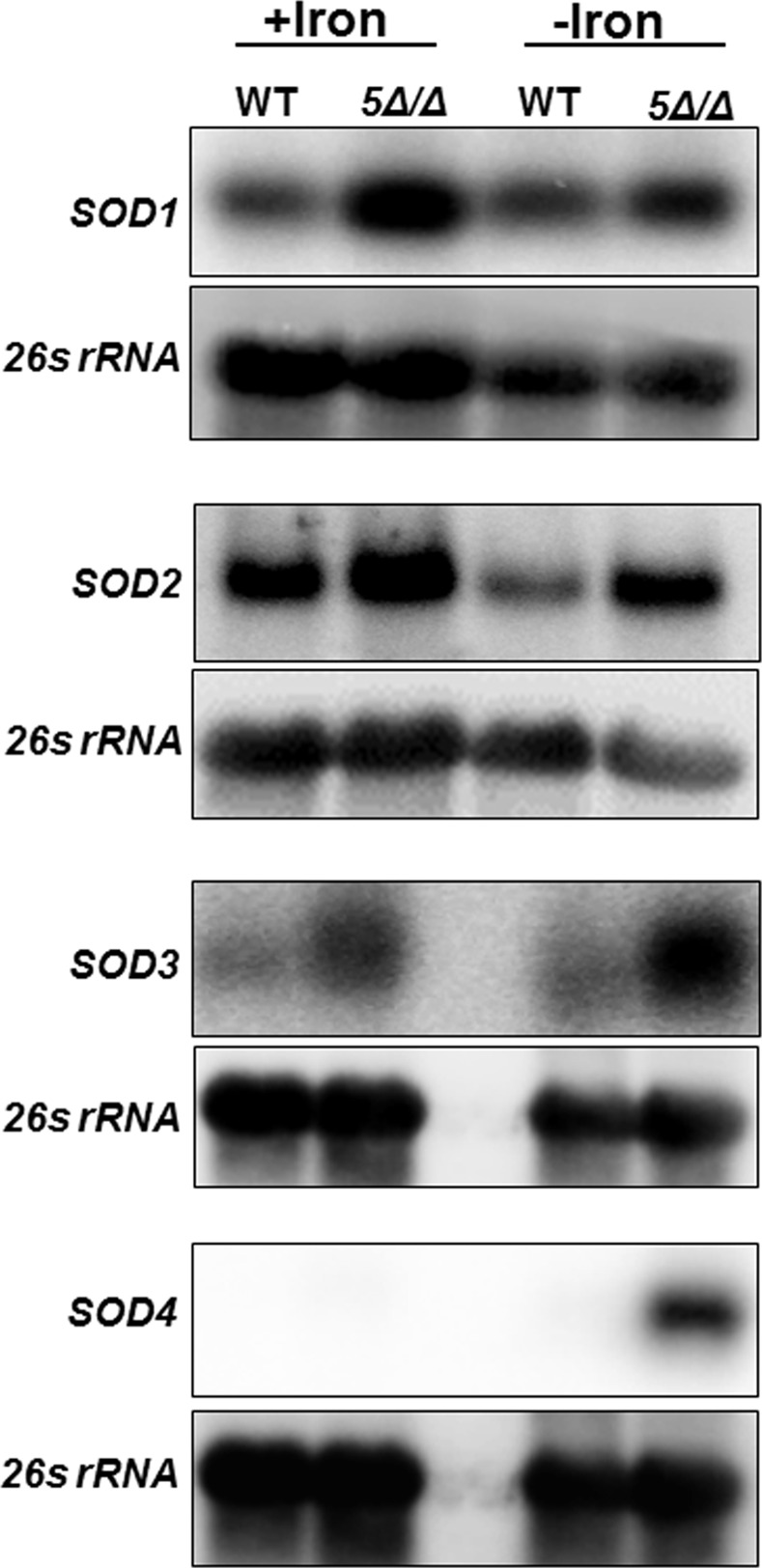
*SOD1*, *SOD2*, *SOD3*, and *SOD4* are regulated by the CCAAT-binding factor. Northern blot analysis was performed to examine the expression of *SOD1*, *SOD2*, *SOD3*, and *SOD4* mRNA as indicated in the wild-type (DMC146) and *hap5Δ/Δ* mutant (DMC117) following growth in iron-replete (+iron) and iron-limiting (-iron) medium. The 26s rRNA was the loading control.

Since the CCAAT-binding factor was involved in the regulation of *CAT1*, it was reasonable to predict that other OSR genes, such as *SOD1-6* (superoxide dismutases), *GRX1-4* (glutaredoxins) and/or *TRX1* (thioredoxin), could be coordinately regulated. We examined the mRNA levels of *SOD1* through *SOD6*, *GRX1*, *GRX2*, *GRX3*, *GRX5* and *TRX1* in the wild type and *hap5Δ/Δ* mutant strains grown in iron replete and iron-limiting YPD medium. We observed that *SOD1*, *SOD2*, and S*OD3* were repressed by the CCAAT-binding factor; however, the repression was not dependent on iron (Fig 2). In contrast, the expression of *SOD4* was only observed under iron-limitation in the *hap5Δ/Δ* mutant, suggesting the CCAAT-binding factor may be involved in the regulation of *SOD4* in an iron-dependent manner. It should also be noted that the mRNA levels of *SOD5* and *SOD6* were examined, but expression of neither was altered by iron availability or the CCAAT-binding factor (not shown).

**Fig 3:**
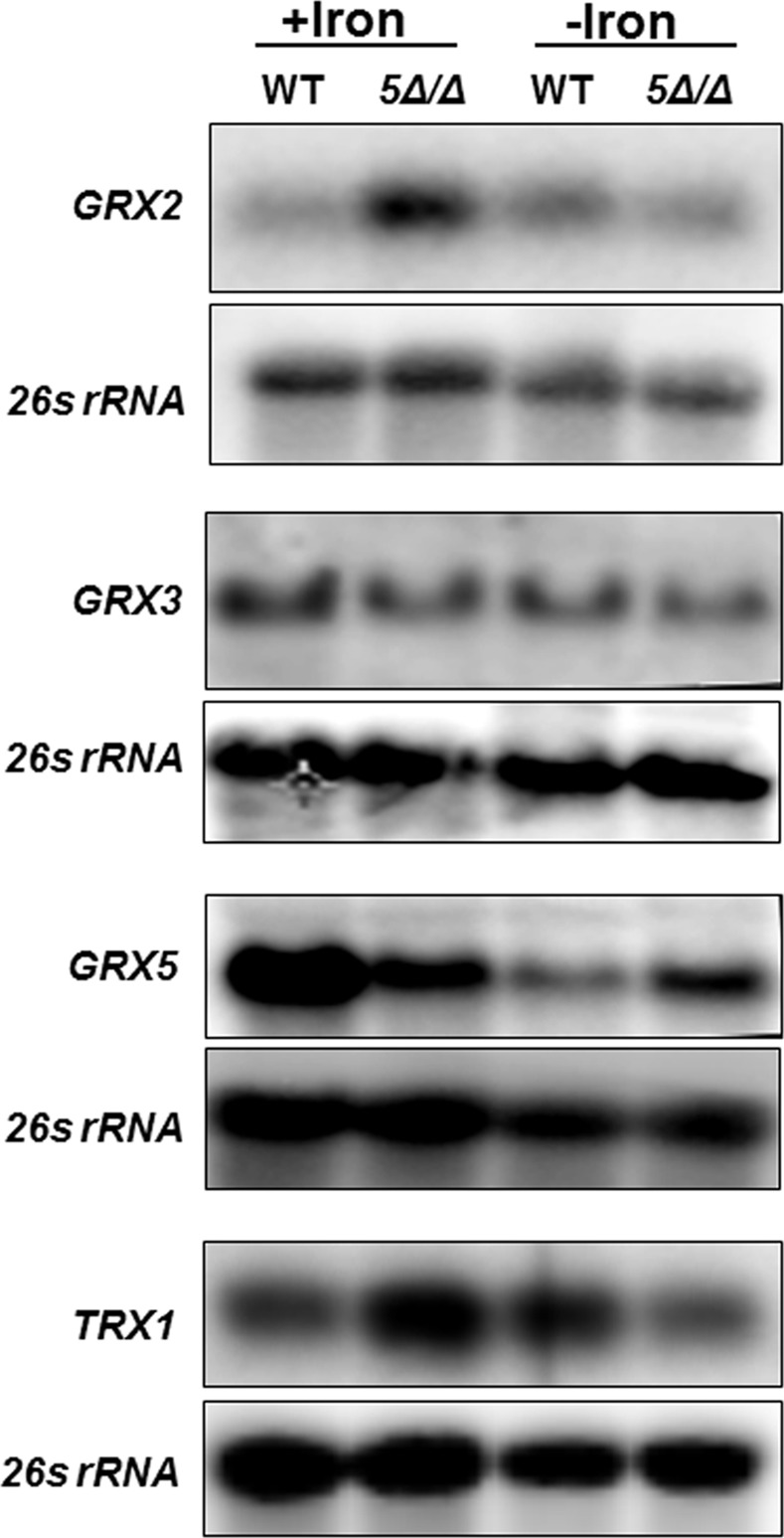
*GRX2*, *GRX5*, and *TRX1* expression is regulated by the CCAAT-binding factor. Northern blot analysis was performed to examine the expression of *GRX2*, *GRX3*, *GRX5*, and *TRX1* mRNA as indicated in the wild-type (DMC146) and *hap5Δ/Δ* mutant (DMC117) following growth in iron-replete (+iron) and iron-limiting (-iron) medium. The 26s rRNA was the loading control.

With respect to the expression of the genes encoding the glutaredoxins and thioredoxin, it was found that *GRX2* and *TRX1* appear to be repressed by the CCAAT-binding factor in iron-replete medium; however, no clear iron-dependent regulation was observed (Fig 3). *GRX3* mRNA expression was unchanged in the *hap5Δ/Δ* mutant or by the iron status of the growth medium. In contrast, *GRX5* demonstrated a pattern of mRNA expression reminiscent of *CAT1*, indicating the CCAAT-binding factor is involved in the activation of *GRX5* during iron-replete growth and repression when iron was limiting (Fig 3), indicating *GRX5* transcription is coordinately regulated with *CAT1*. Although we find both genes have a similar CCAAT-binding factor-dependent response to iron availability, it remains to be shown that the regulation involves the direct binding of the transcription factor to its cognate promoter sequence. The *GRX1* expression was also examined, but no mRNA was detected under the growth conditions used in this study (not shown).

### Role of Hap31 and Hap32 in the iron-dependent regulation of *CAT1*

**Fig 4:**
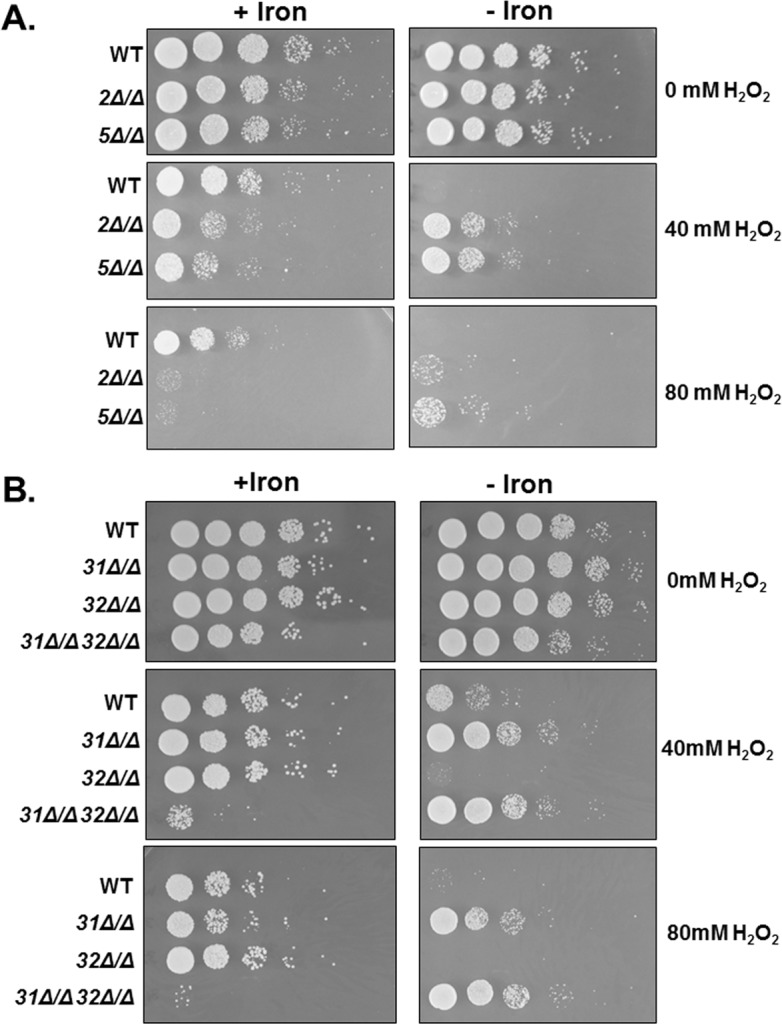
Hap31 and Hap32 display a differential response to hydrogen peroxide stress. **(A)** *C*. *albicans* wild-type (DMC146), *hap2Δ/Δ* (DMC249), and *hap5Δ/Δ* mutant (DMC117), were grown in iron-replete (+iron) or iron-limited (-iron) medium and subsequently exposed to hydrogen peroxide at the indicated concentrations for 2 h at 30°C. Ten-fold serial dilutions were spotted to YPD medium and incubated at 30°C for 2 days. **(B)** *C*. *albicans* wild-type (DMC146), *hap31Δ/Δ* (DMC280), *hap32Δ/Δ* (DMC285), and the *hap31Δ/Δ hap32Δ/Δ* (DMC290) mutants were grown in iron-replete (+iron) or iron-limited (-iron) medium and subsequently exposed to hydrogen peroxide at the indicated concentrations for 2 h at 30°C for 3 days.

The CCAAT-binding factor appears to function in the regulation of several OSR genes; however, the expression of *CAT1* and *GRX5* mRNA in response to iron availability suggested the CCAAT-binding factor may function in transcriptional activation and repression in response to environmental conditions. We wanted to further explore the mechanism behind this dual function. *C*. *albicans* encodes two different Hap3 subunits, termed Hap31 and Hap32, and three putative Hap4 subunits, designated Hap41, Hap42, and Hap43. One putative model to explain the dual role of this multi-subunit transcription factor involves the different Hap3 and/or Hap4 subunits interacting with Hap2 and Hap5 in a combinatorial manner to form distinct CCAAT-binding complexes that either promote or repress transcription in response to environmental signals. To explore this possibility, we examined the oxidative stress sensitivity of various null mutants of the CCAAT-binding factor subunits and compared them to the *hap5Δ*/Δ mutant. Thus, the *hap2Δ/Δ*, *hap31Δ/Δ*, *hap32Δ/Δ*, and *hap31Δ/Δ hap32Δ/Δ* double mutant were grown in iron replete or iron-limiting medium (YPD or YPD + BPS, respectively) and subsequently exposed to various concentrations of hydrogen peroxide and serial dilutions plated on YPD medium (Fig 4). As expected, the *hap2Δ/Δ* strain mimicked the phenotype of the *hap5Δ/Δ* mutant (Fig 4A), with higher sensitivity to oxidative stress than the wild-type after iron-replete growth, yet more resistant to oxidative killing following iron-limited growth. When the *hap31Δ/Δ* and *hap32Δ/Δ* mutants were examined, neither demonstrated increased sensitivity to oxidative stress after iron replete growth (Fig 4B); however, the *hap31Δ/Δ hap32Δ/Δ* double mutant showed sensitivity equivalent to the *hap2Δ/Δ* and *hap5Δ/Δ* mutants. After iron-limited growth, the *hap31Δ/Δ* and the *hap31Δ/Δ hap32Δ/Δ* double mutant demonstrated increased resistance to oxidative stress, analogous to the *hap2Δ/Δ* and *hap5Δ/Δ* mutants, suggesting the loss of Hap31 is key to the oxidative stress resistance observed during iron-limiting growth. Moreover, it was observed that the *hap32Δ/Δ* mutant was more sensitive than the wild-type strain to oxidative stress after growth under iron limitation. These data suggest a relationship between *HAP31* and *HAP32* in which *hap31Δ/Δ* is epistatic to *hap32Δ/Δ* in iron-limiting conditions since the double mutant shows the same survival phenotype to the hydrogen peroxide treatment as the *hap31Δ/Δ* mutant. The epistatic relationship among these genes would indicate a cross-regulatory mechanism between the subunits depending of which Hap3 subunit is associated with the CCAAT-binding factor.

**Fig 5:**
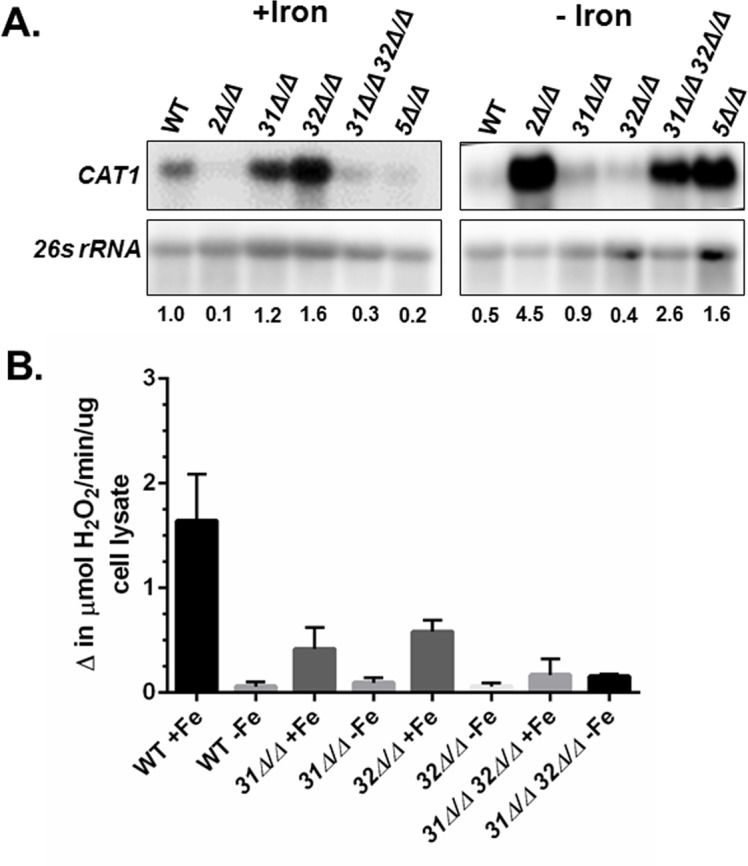
Hap31 and Hap32 are interchangeable in the function of the CCAAT-binding factor. **(A)** Northern blot analysis of *CAT1* mRNA expression in the wild-type (DMC146) and the indicated *hap* mutants following growth in iron-replete (+iron) and iron-limiting (-iron) medium. The 26s rRNA was the loading control. mRNA levels were normalized to the 26s rRNA control using the WT as the reference value. **(B)** Catalase activity in cell extracts derived from the wild type (DMC146), *hap31Δ/Δ* (DMC280), *hap32Δ/Δ* (DMC285), and *hap31Δ/Δ hap32Δ/Δ* (DMC290) mutants following growth in iron-replete (+iron) and iron-limiting (-iron) medium. The enzymatic assays are the average of three independent experiments with the error bars indicating the standard error.

When the *CAT1* mRNA levels were examined by Northern blotting in the various *hap* mutants, it was found that the *hap2Δ/Δ* and *hap31Δ/Δ hap32Δ/Δ* mutants showed a decrease in expression analogous to the *hap5Δ/Δ* after iron replete growth, confirming the loss of CCAAT-binding activity reduced the activation of *CAT1* (Fig 5A). Moreover, the *hap2Δ/Δ* and *hap31Δ/Δ hap32Δ/Δ* mutants demonstrated a similar loss of *CAT1* repression after growth under iron limitation, demonstrating the loss of repression is due to the lack of CCAAT-binding activity. Interestingly, neither the *hap31Δ/Δ* or *hap32Δ/Δ* mutants showed a major decrease in *CAT1* mRNA after iron-replete growth. In fact, the *hap32Δ/Δ* mutant displayed a three to four-fold higher level of expression than the wild-type strain. When the same strains were grown in iron-limiting medium it was found that the *hap31Δ/Δ* and *hap32Δ/Δ* mutants displayed expression similar to the wild-type strain (Fig 5A). To correlate the phenotype of the *hap* mutants with the catalase activity, we examined the catalase activity of the *hap31Δ/Δ*, *hap32Δ/Δ*, and *hap31Δ/Δ hap32Δ/Δ* mutants after growth in iron-replete or iron-limiting conditions. As shown in Fig 5B, the *hap31Δ/Δ hap32Δ/Δ* mutant demonstrated a 6-fold reduction in catalase activity that mimicked the *hap5Δ/Δ* mutant, while the *hap31Δ/Δ* and *hap32Δ/Δ* mutants showed only a 2.5- to 3.5-fold decrease in catalase activity. It seems plausible that the catalase activity, although reduced in the *hap3* mutants, may remain sufficient for the oxidative stress phenotype comparable to wild-type strain (see Fig 4B). After growth in iron-limiting medium, the lysate from the *hap31Δ/Δ hap32Δ/Δ* mutant showed catalase activity that was reproducibly higher than the wild-type strain and similar to the *hap5Δ/Δ* mutant. The residual activity may explain the relative oxidative stress resistance of these strains during iron limitation. The catalase activity of the *hap31Δ/Δ* and *hap32Δ/Δ* single mutants observed after growth in iron-limiting conditions was consistently similar to the wild-type strain, yet the *hap31Δ/Δ* mutant was phenotypically more resistant to oxidative stress under iron limitation and the *hap32Δ/Δ* mutant was more sensitive. We did observe that the catalase activity of the *hap31Δ/Δ* mutant was reproducibly higher than the wild-type strain after iron-limiting growth, conversely the *hap32Δ/Δ* mutant was consistently less than the wild-type; however, these differences were not statistically significant. Nevertheless, there may be a minimum threshold of catalase activity necessary for oxidative stress resistance during iron-limiting growth and the *hap31Δ/Δ* mutant surpasses that minimum while the *hap32Δ/Δ* is below the activity necessary for survival of the oxidative stress conditions used in this study. Alternatively, other OSR genes induced by iron starvation may contribute to the enhanced hydrogen peroxide resistance in a *hap31Δ/Δ* mutant.

### Hap43 is solely responsible for the iron-dependent regulation of *CAT1*

**Fig 6:**
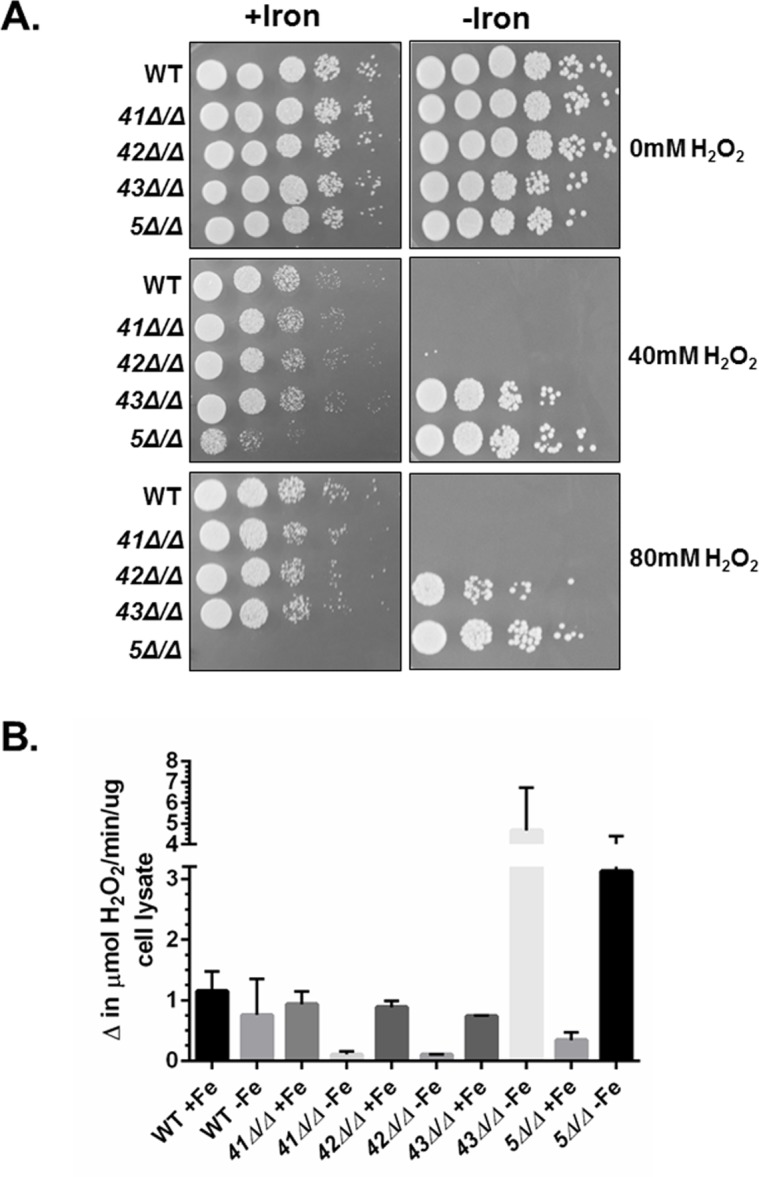
The role of the individual Hap4-like subunits in regulating the OSR. **(A)** *C*. *albicans* wild-type (DMC146), *hap41Δ/Δ* (DMC190), *hap42Δ/Δ* (DMC350), *hap43Δ/Δ* (DMC351) and *hap5Δ/Δ* mutant (DMC117) were grown in iron-replete (+iron) or iron-limited (-iron) medium and subsequently exposed to hydrogen peroxide at the indicated concentrations for 2 h at 30°C. Ten-fold serial dilutions were spotted to YPD medium and incubated at 30°C for 3 days. **(B)** Catalase activity assays were performed on extracts from the wild type (DMC146), *hap41Δ/Δ* (DMC190), *hap42Δ/Δ* (DMC350), *hap43Δ/Δ* (DMC351) and *hap5Δ/Δ* mutant (DMC117) mutants following growth in iron-replete (+iron) and iron-limiting (-iron) medium. The enzymatic assays are the average of three independent experiments with the error bars indicating the standard error.

*C*. *albicans* is predicted to encode three distinct Hap4-like proteins, termed Hap41, Hap42, and Hap43 [35]. Previous studies have demonstrated that Hap43, as a subunit of the CCAAT-binding factor, is involved in the iron-dependent regulation of multiple genes [25,26,34,37]. We examined the role of the three Hap4 subunits in the OSR of *C*. *albicans*. We generated homozygous gene deletions of *HAP41*, *HAP42*, and *HAP43* and subsequently examined their sensitivity to hydrogen peroxide after growth in iron replete and iron-limiting medium. As shown in Fig 6A, none of the *hap4* null mutants showed increased sensitivity to hydrogen peroxide stress relative to the wild-type control following iron replete growth, whereas the *hap5Δ*/*Δ* control was highly sensitive. By contrast, the mutants evaluated for oxidative stress sensitivity following iron-limited growth, demonstrated that only the *hap43Δ*/*Δ* mutant had resistance comparable to the *hap5Δ/Δ* strain (Fig 6A). This phenotype indicates that the *hap43Δ/Δ* and *hap5Δ/Δ* mutants should have higher catalase activity after iron-limited growth compared to the wild-type, *hap41Δ/Δ*, and *hap42Δ*/Δ strains. To confirm this prediction, catalase assays were performed on lysates prepared from each strain following iron replete and iron-limiting growth, and the data confirmed that the *hap43Δ/Δ* mutant had significantly higher catalase activity, comparable to that of the *hap5Δ/Δ* mutant (Fig 6B). On the basis of these data, the Hap4 subunits do not appear to play a significant role in the regulation of *CAT1* during iron replete growth; however, Hap43 was needed for the repression of *CAT1* during growth under iron limitation. As a corollary, Hap41 and Hap42 do not appear to function in the regulation of *CAT1*.

**Fig 7:**
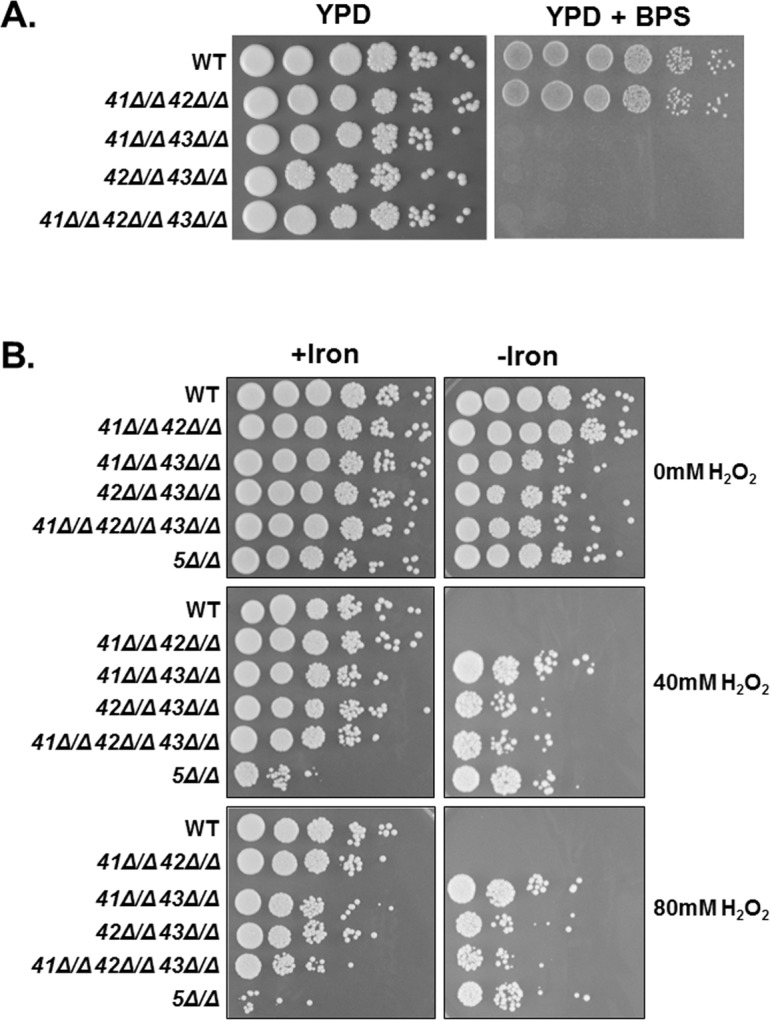
Hap43 is the sole Hap4 subunit involved in the CCAAT-binding factor-mediated oxidative stress response. **(A)** *C*. *albicans* wild-type (DMC146) and indicated *hap4* combination mutants were grown in iron-replete (YPD) or iron-limited (YPD+BPS) liquid medium and ten-fold serial dilutions were spotted to YPD or YPD+BPS, respectively, and incubated at 30°C for 3 days. **(B)** *C*. *albicans* wild-type (DMC146), *hap5Δ/Δ* (DMC117) and indicated *hap4Δ/Δ* combination mutants were grown in iron-replete (+iron) or iron-limited (-iron) medium and subsequently exposed to hydrogen peroxide at the indicated concentrations for 2 h at 30°C. Ten-fold serial dilutions were spotted to YPD medium and incubated at 30°C for 3 days.

The experiments above indicate that Hap43 is solely responsible for the iron-dependent oxidative stress phenotype. To further validate this finding, we constructed strains that included all *hap4* mutant combinations, including the *hap41Δ/Δ hap42Δ/Δ hap43Δ/Δ* triple mutant, to determine whether the different Hap4-like proteins could provide compensatory activity and mask potential phenotypes. Previous studies have demonstrated that the *hap43Δ/Δ* is unable to grow under iron limitation [26,34,36]. We initially examined the growth of the various *hap4* mutants for a growth defect on iron-deficient medium. As shown in Fig 7A, only the mutants that included the *hap43Δ/Δ* knockout were unable to grow on iron-limiting medium, whereas the *hap41Δ/Δ hap42Δ/Δ* double mutant grew comparable to wild type. These data imply that Hap43 is solely important for growth during iron limitation.

To examine the oxidative stress phenotype of the various *hap4* mutants, the strains were grown in iron replete or iron-limiting medium and subsequently exposed to hydrogen peroxide stress. Serial dilutions of the cells were spotted to rich medium and the survival phenotype examined. When grown in iron replete medium, none of the *hap4* null mutants displayed a phenotype that differed significantly from the wild-type strain (Fig 7B), suggesting none of the Hap4 subunits function in the regulation of general OSR in a nutrient-rich environment. In contrast, the iron-limiting growth resulted in significant oxidative stress resistance of strains that contained the *hap43Δ/Δ* mutation (Fig 7B). Importantly, given that the *hap41Δ/Δ hap42Δ/Δ* mutant was phenotypically sensitive to oxidative stress comparable to the wild-type strain, these data strongly support the conclusion that Hap43 is the sole subunit critical for the OSR as well as the repression of genes under iron deficient conditions.

**Fig 8:**
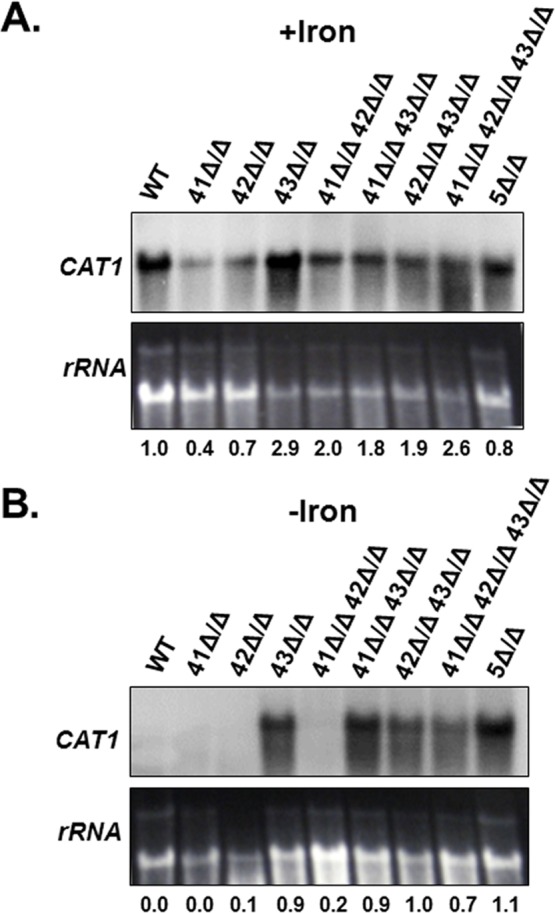
Hap43 is necessary for the CCAAT-binding factor-mediated regulation of *CAT1* in response to iron. Northern blot analysis of *CAT1* mRNA expression in the wild-type (DMC146), *hap5Δ/Δ* mutant (DMC117) and the indicated *hap4Δ/Δ* single or combination mutants following growth in **(A)** iron-replete (+iron) and **(B)** iron-limiting (-iron) medium. The rRNA was the loading control. mRNA levels were normalized to the rRNA control using the WT as the reference value.

Given the phenotypic observations described above for the various *hap4* mutants, we hypothesized that the *CAT1* mRNA levels would not vary significantly after growth in iron replete medium. As a corollary, we predicted the loss of *CAT1* repression during iron-limiting growth in mutants that included the *hap43Δ/Δ* mutation. To confirm this prediction, we performed Northern blots on RNA isolated from all of the *hap4* knockout mutants grown in iron replete and iron-limiting conditions (Fig 8). Although there was some variability in *CAT1* expression in the *hap4* knockout mutants or combination mutants after growth in iron replete medium (Fig 8A), we did not observe statistically significant differences that were reproducible in three independent Northern blot experiments. This led to the conclusion that none of the Hap4 subunits played a significant role in *CAT1* regulation in rich growth medium. In contrast, the expression of *CAT1* after iron-limiting growth clearly demonstrated that Hap43 was important for the repression of *CAT1* during iron deprivation since the *hap43Δ/Δ* mutant as well as any combination mutant that included *hap43Δ/Δ* displayed the loss of *CAT1* repression (Fig 8B). These data unambiguously support the role of Hap43 as the effector subunit that interacts with the CCAAT-binding factor to regulate genes involved in the OSR in response to iron.

## Discussion

The CCAAT-binding factor has been previously shown to be a transcriptional regulator involved in modulating the expression of genes involved in respiratory metabolism [35] and iron acquisition/utilization [25,26,34,36,55]. In addition, prior microarray expression studies have implicated the CCAAT-binding factor in the iron-dependent regulation of genes involved in oxidative stress [34]. In this work we further dissect the requirement of each of the CCAAT-binding factor subunits for the regulated expression of some OSR genes in response to iron. In addition, we connect the mRNA expression of *CAT1* to the *in vivo* activity of catalase, measured by the cell’s ability to overcome oxidative stress. Finally, we present data suggesting the modular nature of the CCAAT-binding complex, with the Hap31 or Hap32 subunits, with or without Hap43, is essential for the assembly of the transcription complexes required for either activation or repression of the indicated OSR genes.

### The CCAAT-binding factor has a dual and contrasting role in the regulation of OSR genes in response to iron

During cell metabolism, reactive oxygen species are generated primarily through the mitochondrial electron transport chain via the partial reduction of oxygen through the transfer of one, two, or three electrons, generating superoxide, hydrogen peroxide or hydroxyl radicals. *C*. *albicans*, like other eukaryotes, has developed antioxidant mechanisms such as superoxide dismutases, catalase, thioredoxins and glutaredoxins to neutralize the reactive oxygen species and repair cell damage. We hypothesized that the CCAAT-binding factor may play a central role in regulating the oxidative stress response in *C*. *albicans*, thereby coordinately regulating iron acquisition/utilization, respiratory metabolism and the oxidative stress response.

In our initial gene expression analysis of *CAT1* we found the mRNA levels decreased to a basal level in the *hap5Δ/Δ* strain as compared to the wild-type, implicating the CCAAT-binding factor in transcriptional activation. However, during iron deficient growth the expression levels reversed; with the wild-type expression nearly null and the *hap5Δ/Δ* strain showing significant *CAT1* mRNA, implicating the CCAAT-binding factor in transcriptional repression. Moreover, the luciferase activity of the *CAT1-Rluc* promoter fusion shows that the *CAT1* promoter drives gene expression in a CCAAT-binding factor-dependent manner. These data demonstrate the role of the CCAAT-binding factor as an activator or repressor of target genes in response to iron. Importantly, these findings complement previous data demonstrating the role of this transcription factor in the regulation of respiratory genes in response to carbon source availability [35]. Moreover, this work is consistent with previous reports that have shown that the CCAAT-binding factor functions as a transcriptional activator/repressor of numerous genes encoding proteins that utilize iron as a cofactor in response to iron availability [25,26,34].

This finding prompted us to ask whether the CCAAT-binding factor also regulates other OSR genes in *C*. *albicans*. Among the six genes predicted to encode superoxide dismutase enzymes, three of them, *SOD1*, *SOD2*, and *SOD3*, were repressed by the CCAAT-binding factor, but in an iron-independent manner, as the transcript levels increased in the *hap5Δ/Δ* strain compared to the wild-type under iron-replete as well as iron-limiting conditions. The CCAAT-binding factor-dependent repression of *SOD2* and *SOD3* has also been reported by microarray studies used to compare the transcriptional profiling of a wild-type to a *hap43* (*cap2*) mutant under iron limitation [34]. Since Hap43 is an effector subunit of the CCAAT-binding factor [25,26,34,36], one would predict that null mutations in the genes encoding the other Hap subunits would display a similar profile. Both cytoplasmic Sod1 and Sod3 have been shown to be critical for *C*. *albicans* virulence although they are induced under different growth conditions, consistent with the low expression levels we observed in exponentially grown wild-type cells. Sod1 is known to be induced under hyphal-promoting conditions and it has been implicated in protection against ROS generated by menadione and macrophages [21,56]. Sod1 expression is repressed during stationary phase as a defense mechanism to evade copper toxicity [21,22]. Sod3 is an unusual cytoplasmic MnSod expressed during stationary phase [57]. The transcript level of the mitochondrial Sod2 was most abundant in the wild-type cells under iron-replete conditions, an observation consistent with the protective role of Sod2 against intracellular superoxide anions. The iron replete growth conditions used in our study would yield normal ROS levels as a byproduct of respiratory metabolism [22]. In agreement with this response, *SOD2* mRNA levels are diminished under iron limitation when ROS levels would be reduced. Indeed, exposure to hydrogen peroxide does not seem to increase expression of *SOD2*, emphasizing its role in intracellular ROS detoxification [15]. No visible transcripts were detected for the cell surface associated Sod4, Sod5 and Sod6 in iron replete conditions. This finding is consistent with the role of these superoxide dismutases in the detoxification of the extracellular ROS threat, especially those generated by the host macrophages and neutrophils [58]. However, under iron deprivation we observed a strong CCAAT-binding factor-dependent repression of *SOD4*, as evidenced by high mRNA levels present in the *hap5Δ/Δ* strain. A connection between superoxide stress and intracellular iron levels has been proposed in *S*. *cerevisiae*, where mutations in superoxide dismutase genes show altered iron homeostasis [59,60]. Thus, it is possible that a similar mechanism exists in *C*. *albicans*, where the repression of the *SOD* genes by the CCAAT-binding factor during iron limitation permits the mobilization of iron to more essential processes.

We found that two of the glutaredoxin-encoding genes, *GRX2* and *GRX5*, are regulated by the CCAAT-binding factor in a contrasting manner. While the CCAAT-binding complex appeared to repress *GRX2*, *GRX5* was activated by the transcription factor in iron-replete medium. In contrast, during iron limiting growth *GRX2* expression appears to be CCAAT-binding factor-independent; while *GRX5* was repressed in a manner similar to *CAT1*. Grx2 has been implicated in the resistance to PMN-mediated killing by the host [23,61]. On the other hand, the Grx5 ortholog in *S*.*cerevisiae* is a mitochondrial matrix protein involved in the incorporation of the Fe-S clusters into respiratory chain proteins [61]. Thus, on the basis of cellular function, the CCAAT-binding factor seems to regulate *GRX2* and *GRX5* expression differentially in the same environmental conditions. *GRX5* repression under iron limitation may contribute to increasing intracellular iron, and mobilizing it to essential proteins needed for cell survival. In support of this hypothesis, it has been shown that a *S*. *cerevisiae grx5Δ* strain accumulates iron intracellularly [11,61].

We also observed the CCAAT-binding factor-dependent expression of the thioredoxin gene *TRX1* in response to iron; however, it was opposite of that seen with *CAT1* and *GRX5*. The repression and activation observed during iron replete and iron limiting growth, respectively, may represent an unexplored function of Trx1 related to iron metabolism. It should be noted that this pattern of *TRX1* regulation was also reported in a whole genome transcriptional profile that compared a wild-type to a *hap43Δ/Δ* mutant under iron limited growth [34]. In *S*. *cerevisiae* it has been shown that the thioredoxins and glutaredoxins are relevant for maintaining the cellular thiol-redox system; however, evidence suggests that they operate through different non-redundant pathways [62,63]. For *C*. *albicans*, additional work is needed to understand the contribution and regulation of these genes in the OSR and iron homeostasis. What is clear is that the CCAAT-binding factor can specifically activate as well as repress some of these genes in response to the iron available during cell growth.

### The CCAAT-binding factor regulates the in vivo OSR

The hydrogen peroxide sensitivity assay allowed us to examine the cellular response of *C*. *albicans* when confronted with ROS after prior growth in iron-replete or iron deficient environments. The survival to peroxide treatment correlated with *CAT1* mRNA levels and the catalase activity of cells in response to iron. Moreover, the assay demonstrated that the integrity of the CCAAT-binding factor is essential for *C*. *albicans* to cope with peroxide stress. This is supported by the fact that any one of the strains carrying a deletion of the subunits essential for the integrity of the CCAAT-binding factor, *i*.*e*., *hap2Δ/Δ*, *hap5Δ/Δ*, or the double deletion *hap31Δ/Δ hap32Δ/Δ*, was unable to survive the hydrogen peroxide treatment if the cells were previously grown in iron-replete medium. The opposite was seen when the cells were grown in iron-limited conditions, in perfect agreement with the high *CAT1* mRNA levels as well as the catalase activity of the respective strains. This is consistent with prior studies that have shown that the CCAAT-binding factor functions as a transcriptional repressor of numerous genes that utilize iron as a cofactor after exposure to iron-limiting growth conditions [25,26,34]. Moreover, the OSR has been shown to be regulated by the CCAAT-binding factor in the filamentous fungus, *Aspergillus nidulans*. In *A*. *nidulans*, the complex is involved in redox sensing via the oxidative modification of thiol groups in the evolutionarily conserved cysteine residues of the histone fold motif of HapC, which results in the regulation of OSR genes [64]. The HapC orthologs in *C*. *albicans*, Hap31 and Hap32, also share the conserved cysteine residues, suggesting a possible mechanism for their participation in the OSR. Since either protein could serve as a putative sensor of the redox state of the cell, it is plausible that their function is dependent on environmental cues.

### The modular nature of the CCAAT-binding complex is essential for the differential regulation of *CAT1* in response to iron

Since the CCAAT-binding factor serves contrasting roles as an activator or repressor in response to iron, we hypothesized that this may be achieved through the differential recruitment of the Hap31 or Hap32 as well as Hap41, Hap42, or Hap43 to form functional CCAAT-binding complexes. Our data showed that the presence of either Hap31 or Hap32 is absolutely essential for the activation of *CAT1* in iron-replete conditions, since the strain lacking both genes had less catalase and recovered poorly from peroxide stress, mimicking the *hap5Δ/Δ* mutant. Thus, it appears that both subunits are expressed and capable of compensating for each other in the formation and function of the complex. During iron-limited growth the regulation of *CAT1* is more complex. The mRNA levels indicate that the presence of either Hap31 or Hap32 represses *CAT1* expression to similar levels and comparable to the wild-type levels, again suggesting a compensatory role between these subunits in the formation of an active CCAAT-binding complex. However, in spite of the low levels of catalase the *hap31Δ/Δ* strain showed resistance to the hydrogen peroxide treatment. This unexpected result may reflect threshold levels of catalase sufficient for the protective function, or alternatively, a catalase-independent mechanism of managing peroxide stress that was only manifested in a *hap31Δ/Δ* mutant. Such a mechanism could involve other OSR genes that are expressed under iron limitation. The latter possibility has been suggested in *C*. *glabrata* where a *cta1Δ* (*cat1Δ*) strain remains capable of adaptation to oxidative stress [65].

Our analysis of *CAT1* expression and the survival to peroxide stress in the strains containing single, double, and triple combination of *hap41Δ/Δ*, *hap42Δ/Δ* and *hap43Δ/Δ* alleles lead to the conclusion that none of the Hap4-like subunits were necessary for expression during iron-replete growth. In contrast, during iron limitation, Hap43 was the sole Hap4-like subunit responsible for *CAT1* repression. This is consistent with the fact that *C*. *albicans* Hap43 has been reported to be a global repressor of genes encoding proteins that involve utilization of iron in iron-limiting environments; a mechanism that requires the physical interaction between Hap43 and the Hap5 subunit of the CCAAT-binding complex [34]. Evolutionarily, the Hap43 orthologs from other yeast and fungi, including HapX (*A*. *nidulans*, *Aspergillus fumigatus* and *Cryptococcus neoformans*) and Php4 (*Schizosaccharomyces pombe*), have been implicated in the regulation of genes involved in iron transport/utilization [66–69]. Hap43 and HapX share three cysteine-rich protein domains, which have been proposed to mediate iron sensing through the formation of an iron-binding domain [66,70]. Whether this domain coordinates iron or iron-sulfur clusters remains to be established.

One of these genes regulated by the CCAAT-binding factor in *C*. *albicans* is *CYC1*, encoding cytochrome c, which is repressed during iron limitation in a Hap5- and Hap43-dependent manner and activated by the CCAAT-binding factor under iron-replete conditions [51]. This is relevant because previous studies have demonstrated that *CYC1* is regulated by the CCAAT-binding factor in a carbon source-dependent manner [35]. Thus, the CCAAT-binding factor serves as a modulator of gene expression in response to both carbon source and iron. Our data indicates that the CCAAT-binding factor regulates the OSR genes via its regulation of iron uptake and utilization; whereas, Hog1 and Cap1 have previously been shown to be direct transcriptional regulators of the OSR genes [16,71–73]. Importantly, fluctuations in the level of intracellular iron have a direct impact on the redox potential within the cell. It makes sense that a multi-subunit transcription factor could sense both redox status and the availability of iron within the cell, and coordinate gene expression accordingly. Therefore, it will be interesting to investigate whether such a coordinated response is regulated in *C*. *albicans* by the potential redox sensing via Hap31 and/or Hap32 along with the putative iron-sensing through the cysteine-rich domains of Hap43.

Why is it advantageous to co-regulate genes involved in respiration, iron uptake/utilization, and the OSR? First, the human host is essentially a low iron environment due to sequestration of iron with proteins such as transferrin, ferritin, lactoferrin as well as other iron-binding proteins [28–30]. While *C*. *albicans*, like many other pathogens, has evolved multiple sophisticated mechanisms for scavenging iron from the human host [31,32], the organism must adjust its metabolic needs to meet this challenge. Moreover, dependent on the specific micro-environment in the host, the availability of iron can vary dramatically [25,28]. To meet this metabolic challenge, *C*. *albicans* uses the CCAAT-binding factor to control the expression of genes involved in iron acquisition/utilization, respiration, and the OSR. Although our *in vitro* studies do not aim to mimic the host-pathogen interaction, one can envision the iron-limited environment of the human host activating all the fungal iron-scavenging mechanisms, while repressing OSR genes and genes that express iron-requiring proteins. However, once adequate iron levels have been established, *C*. *albicans* can quickly express OSR genes and modulate a strong response to the host defense mechanisms, such as the oxidative burst following phagocytosis by neutrophils.

## Supporting Information

S1 Table *Candida albicans* strains used in this study. (DOCX)

S2 Table Oligonucleotides used in this study. (DOCX)
