# Supplementary material for: The Iron-Dependent Regulation of the Candida albicans Oxidative Stress Response by the CCAAT-Binding Factor
Source: PLoS One. 2017 Jan 25;12(1):e0170649. doi: 10.1371/journal.pone.0170649 (PMC5266298; doi:10.1371/journal.pone.0170649)
Supplement: S1 Table — (DOCX) [file pone.0170649.s001.docx]

**S1 Table. *Candida* *albicans* strains used in this study.**

| **Strain** | **Genotype** | **Reference** |
| --- | --- | --- |
| **BWP17** | *ura3Δ::imm434/ura3Δ::imm434 his1Δ::hisG/his1Δ::hisG arg4Δ::hisG/arg4Δ:hisG* | [47] |
| **DMC108** | *ura3Δ::imm434/ura3Δ::imm434 his1Δ::hisG/his1Δ::hisG arg4Δ::hisG/arg4Δ:hisG hap5Δ::URA3/hap5Δ::HIS1* | [35] |
| **DMC117** | *ura3Δ::imm434/ura3Δ::imm434 his1Δ::hisG/his1Δ::hisG arg4Δ::hisG/arg4Δ:hisG-ARG4 hap5Δ::URA3/hap5Δ::HIS1* | [35] |
| **DMC146** | *ura3Δ::imm434/ura3Δ::imm434 his1Δ::hisG/his1Δ::hisG-HIS1 arg4Δ::hisG/arg4Δ:hisG-ARG4-URA3* | [35] |
| **DMC190** | *ura3Δ::imm434/ura3Δ::imm434 his1Δ::hisG/his1Δ::hisG arg4Δ::hisG/arg4Δ:hisG-ARG4 hap41Δ::URA3/hap41Δ::HIS1* | This study |
| **DMC249** | *ura3Δ::imm434/ura3Δ::imm434 his1Δ::hisG/his1Δ::hisG arg4Δ::hisG/arg4Δ:hisG-ARG4 hap2Δ::URA3/hap2Δ::HIS1* | [51] |
| **DMC280** | *ura3Δ::imm434/ura3Δ::imm434 his1Δ::hisG/his1Δ::hisG arg4Δ::hisG/arg4Δ:hisG-ARG4 hap31Δ::URA3/hap31Δ::HIS1* | [51] |
| **DMC285** | *ura3Δ::imm434/ura3Δ::imm434 his1Δ::hisG/his1Δ::hisG arg4Δ::hisG/arg4Δ:hisG-ARG4 hap32Δ::URA3/hap32Δ::HIS1* | [51] |
| **DMC290** | *ura3Δ::imm434/ura3Δ::imm434 his1Δ::hisG/his1Δ::hisG arg4Δ::hisG/arg4Δ:hisG-ARG4 hap31Δ::hisG/hap31Δ::hisG hap32Δ::URA3/hap32Δ::HIS1* | [51] |
| **DMC350** | *ura3Δ::imm434/ura3Δ::imm434 his1Δ::hisG/his1Δ::hisG-URA3-HIS1 arg4Δ::hisG/arg4Δ:hisG-ARG4 hap42Δ::hisG/hap42Δ::hisG* | This study |
| **DMC351** | *ura3Δ::imm434/ura3Δ::imm434 his1Δ::hisG/his1Δ::hisG-URA3-HIS1 arg4Δ::hisG/arg4Δ:hisG-ARG4 hap43Δ::hisG/hap43Δ::hisG* | This study |
| **DMC352** | *ura3Δ::imm434/ura3Δ::imm434 his1Δ::hisG/his1Δ::hisG-URA3-HIS1 arg4Δ::hisG/arg4Δ:hisG-ARG4 hap42Δ::hisG/hap42Δ::hisG hap43Δ::hisG/hap43Δ::hisG* | This study |
| **DMC353** | *ura3Δ::imm434/ura3Δ::imm434 his1Δ::hisG/his1Δ::hisG arg4Δ::hisG/arg4Δ:hisG-ARG4 hap41Δ::URA3/hap41Δ::HIS1 hap42Δ::hisG/hap42::hisG* | This study |
| **DMC354** | *ura3Δ::imm434/ura3Δ::imm434 his1Δ::hisG/his1Δ::hisG arg4Δ::hisG/arg4Δ:hisG-ARG4 hap41Δ::URA3/hap41Δ::HIS1 hap43Δ::hisG/hap43::hisG* | This study |
| **DMC355** | *ura3Δ::imm434/ura3Δ::imm434 his1Δ::hisG/his1Δ::hisG arg4Δ::hisG/arg4Δ:hisG-ARG4 hap41Δ::URA3/hap41Δ::HIS1 hap42Δ::hisG/hap42Δ::hisG hap43Δ::hisG/hap43Δ::hisG* | This study |
| **DMC356** | *ura3Δ::imm434/ura3Δ::imm434 his1Δ::hisG/his1Δ::hisG-HIS1-URA3 arg4Δ::hisG/arg4Δ:hisG-ARG4-CAT1-Rluc* | This study |
| **DMC357** | *ura3Δ::imm434/ura3Δ::imm434 his1Δ::hisG/his1Δ::hisG arg4Δ::hisG/arg4Δ:hisG-ARG4-CAT1-Rluc hap5Δ::URA3/hap5Δ::HIS1* | This study |
