# Supplementary material for: The Iron-Dependent Regulation of the Candida albicans Oxidative Stress Response by the CCAAT-Binding Factor
Source: PLoS One. 2017 Jan 25;12(1):e0170649. doi: 10.1371/journal.pone.0170649 (PMC5266298; doi:10.1371/journal.pone.0170649)
Supplement: S2 Table — (DOCX) [file pone.0170649.s002.docx]

**S2 Table. Oligonucleotides used in this study.**

| **Name** | **Sequence** |
| --- | --- |
| oDM0343 | 5’-GGCCGGATCCAATTGGTTTAATTTGAATTTTTCAACAATAATCAAC AAG-3’ |
| oDM0344 | 5’-GGCCCTGCAGCTACATACACTTATGACTACAAAGAATACAAATATAG-3’ |
| oDM0345 | 5’-GGCCGGATCCAGTCACGCAAATCATCGATCCAGTATATCTACC-3’ |
| oDM0346 | 5’-GGCCAAGCTTGGGATTAGACATTTGGTTTGGGATTTTGGTAAAGAA TTAACC-3’ |
| oDM0369 | 5’-GGTGAGGCATGAGTTTCTGCTCTCTCA-3’ |
| oDM0370 | 5’-CTGTATATCGGCACCACTCAATAAGTTACAGCA-3’ |
| oDM0382 | 5’-GGCCGGATCCTTGGATGGTATAAACGGAAAC-3 |
| oDM0383 | 5’-GGCCGGATCCAGGACCACCTTTGATTGAAATAG-3’ |
| oDM0384 | 5’-GGCCTGATCACTGGAGGATGAGGAGACAGAAG-3’ |
| oDM0385 | 5’-GGCCTGATCAGGTGTGTACATCAAGGTGGTAG-3’ |
| oDM0394 | 5’-GGCCGGATCCTAAATCTAAACTATTGTAACACTTCTAACCAATCAC-3’ |
| oDM0395 | 5’-GGCCGTCGACCTACTGGTGCTGGTGCAGGTAATACTATTGCTGGTG-3’ |
| oDM0396 | 5’-CAGGAATGCGCAGTAGTTTCCGAAATCGGTC-3’ |
| oDM0459 | 5’-TAGCGGTTCTGACGTGCAAATCGATCGTCG-3’ |
| oDM0460 | 5’-ACTAACACCTTTTGTGGTGTCTGATGAGCG-3’ |
| oDM0588 | 5’-GGCCAGATCTCCGATAATGTTATCATAAACTGATAG-3’ |
| oDM0589 | 5’-GGCCAGATCTGGGTTGCTATTTTGGTTTGATTGGG-3’ |
| oDM0590 | 5’-GGCCAGATCTCCTTTTGCGGGCATGTTGTTCAAATTG-3’ |
| oDM0591 | 5’-GGCCAGATCTGTTTGACTTACAGTATGAAGTATTTCCG-3’ |
| oDM0617 | 5’-GACGCAAATACATGATAACCGAGGGT-3’ |
| oDM0620 | 5’-GGCCACTAGTTCACTCAATTACAATTCCA-3’ |
| oDM0621 | 5’-CGATATCACTGATATTTGTGC-3’ |
| oDM0622 | 5’-GGTACCCAATCTGTGTCTGTGAG-3’ |
| oDM0626 | 5’-CTATCAGATCATCATCTCGTG-3’ |
| oDM0627 | 5’-GCTTCCTTCCAGTTGATAAC-3’ |
| oDM0628 | 5’-GTAAACGGGTACAAATGCCGC-3’ |
| oDM0629 | 5’-GCCTCAGCCCAGTTGATCACG-3’ |
| oDM0630 | 5’-GAATAATTTCCGTGGC-3’ |
| oDM0631 | 5’-GGCTGATTGAACCGTCTC-3’ |
| oDM0632 | 5’-CGAGTCAGAACAACAGTTCAC-3’ |
| oDM0633 | 5’-CCAATCCACCTTGGAATTCACC-3’ |
| oDM0634 | 5’-CACGTTGTCACTGAAG-3’ |
| oDM0635 | 5’-GCTTGTTTAATAGCAGC-3’ |
| oDM0650 | 5’-GAACAAGAATCCGAATCCG-3’ |
| oDM0651 | 5’-GGGTCAAGCCAATGACACC-3’ |
| oDM0652 | 5’-GTTGCTCTTGCATTAGCAGG-3’ |
| oDM0653 | 5’-CACCGGCAATACCAGCAGC-3’ |
| oDM0654 | 5’-GCTTTGGCTGGTGATGCACC-3’ |
| oDM0655 | 5’-CACCTGCTGCCAAGAGACC-3’ |
| oDM0656 | 5’-CAATAGCTGCTTCAGACAAG-3’ |
| oDM0657 | 5’-CCTGCAGCTATTCCCAATAC-3’ |
| oDM0660 | 5’-GGGGCATGCAAAATCCTGGGTCACAGAAATATGG-3’ |
| oDM0661 | 5’-GGGGGGATCCAATAAATAATTTATATATAAATAGG-3’ |
| oDM0665 | 5’-CATCAACAATGGTTTCATCTC-3’ |
| oDM0666 | 5’-GCAGCTTTGATTTTGTCATC-3’ |
